# Complementary Intradermal and Patch Testing for Increased Diagnostic Accuracy of Nickel Allergy in Non-Celiac Wheat Insensitivity

**DOI:** 10.3390/nu9060536

**Published:** 2017-05-24

**Authors:** Brittanya A. Limone, Annelise Rasmussen, Sue Min Kwon, Sharon E. Jacob

**Affiliations:** Department of Dermatology, Loma Linda University, 11370 Anderson Street, Suite 2600, Loma Linda, CA 92354, USA; blimone@llu.edu (B.A.L.); annelise.rasmussen@gmail.com (A.R.); skwon@llu.edu (S.M.K.)

**Keywords:** non-celiac wheat insensitivity, nickel, systemic nickel allergy syndrome, intradermal testing

## Abstract

D’Alcamo et al. astutely highlighted a potential immunologic association between nickel allergy, determined by positive epicutaneous patch testing, and the rise of non-celiac wheat sensitivity (NCWS) in the world of gluten-related diseases. Consecutive algorithms including both patch and intradermal testing could provide vital information to more accurately define the patient populations with NCWS, systemic nickel allergy syndrome, and nickel-associated allergic contact dermatitis.

Dear Editor,

We applaud D’Alcamo et al. for highlighting a potential immunologic association between nickel allergy, determined by positive epicutaneous patch testing in patients experiencing a type IV reaction, and the rise of non-celiac wheat sensitivity (NCWS) in the world of gluten-related diseases [1]. The sensitization prevalence to the ubiquitous metal nickel in the US is estimated to be 19.5% of adults, with a conservatively estimated 0.375% (1.2 million) notably suffering from Ni-associated Allergic Contact Dermatitis (Ni-ACD) [2,3]. Calnan and Wells made a pivotal observation in 1956; while Ni-ACD was already known to affect industrial workers chronically exposed to nickel, they noted a remarkable increase in the incidence associated with popular use of the metal in clothing manufacturing [4]. They noted women were having a primary reaction to the nickel stocking suspenders at the site of skin contact, in addition to flare-ups of eczema systemically at secondary sites not directly in contact with nickel, such as eyelids, earlobes, and elbow flexures [4]. While Ni-ACD typically presents localized to the site of contact with the associated source, the systemic contact dermatitis described by Calnan and Wells and its subset systemic nickel allergy syndrome (SNAS) have been notably reported [5].

SNAS encompasses a spectrum of clinical presentations (respiratory, gastrointestinal, genitourinary, hematologic) that occur once a nickel-sensitized individual has had continuous exposures to nickel through diet, inhalation, or implantation [6,7]. The most common presentations of SNAS include gastrointestinal symptoms (89%) and cutaneous symptoms (52%). Of note, gastrointestinal symptoms in SNAS may present similarly to NCWS with bloating, abdominal pain, nausea with or without vomiting, diarrhea, and constipation. A positive nickel patch test in a patient whose symptoms resolve upon instituting a low nickel diet (and recur with positive nickel oral provocation test) is thought to be highly suggestive of SNAS. Clinical improvement following implementation of low nickel diets where nickel-rich foods are eliminated (e.g., legumes (lentils, peas, beans), tomatoes, nuts, cocoa, oats, and whole wheat) [6] are further indication of the diagnosis [5].

Like SNAS, the patho-etiology of NCWS, as D’Alcamo et al. explains, remains to be elucidated and the precise diagnostic test to confirm remains to be determined. The immediate reaction times and widespread eruptions in SNAS are inconsistent with a classic T-cell mediated, delayed hypersensitivity [5,8] and raise question as to the utility of the epicutaneous patch test in determining SNAS as the underlying cause of symptoms. Furthermore, studies have identified nickel-induced inflammatory environments within SNAS patients’ intestinal mucosa characterized by lymphoplasmacellular inflammatory infiltrate and duodenal villus deformation [6]. It is believed that the altered functioning in the intestinal barrier allows for increased mucosal immune system exposures to antigens and subsequent development of dietary-induced SNAS eruptions [6].

Given the similarities between the symptoms of SNAS and NCWS, the time course for symptomatology, and the altered immune response, it stands to reason that there may be a role for both epicutaneous patch testing (to detect T-cell mediated delayed hypersensitivity reactions) and intradermal testing (to confirm IgE mediated immediate hypersensitivity reactions) in elucidating the mechanisms behind SNAS and NCWS and the pathophysiologic difference between the two. Intradermal (ID) testing may have utility in the setting of uncertain patch tests where the immunologic response may not solely be a type IV reaction, especially in cases that do not exhibit the classic delay in presentation [9,10]. As NCWS patients with confirmed nickel sensitization have been reported to have a higher incidence of atopic disease (AD) versus those with a functional gastrointestinal disorder, such as irritable bowel syndrome [1], it stands to reason that ID testing may prove useful [10,11]. Consecutive algorithms including both patch and ID testing could provide vital information to more accurately define the patient populations with NCWS, SNAS, and Ni-ACD [7].
